# Agile Software Development Practices and Success in Outsourced Projects: The Moderating Role of Requirements Risk

**DOI:** 10.1007/978-3-030-49392-9_4

**Published:** 2020-05-06

**Authors:** Oliver Krancher

**Affiliations:** 6 University of Oslo, Oslo, Norway; 7 Monash University, Clayton, VIC Australia; 8 IT University of Copenhagen, Copenhagen, Denmark; 9 University of British Columbia, Vancouver, BC Canada; IT University of Copenhagen, Rued Langgaards Vej 7, 2300 Copenhagen, Denmark

**Keywords:** Agile software development, Agile practices, Requirements risk, Project success, Continuous integration, Continuous analysis, Joint decision making, Agile requirements engineering

## Abstract

Although agile practices are gaining in popularity, there is little evidence showing how particular agile practices, in particular those involving the client, affect the success of outsourced software projects. Data from a matched survey of sponsors and developers in 60 outsourced information systems projects indicate negative effects of continuous analysis and positive effects of joint decision making and continuous integration on project success. Moreover, interaction analyses show that some positive effects are enhanced and negative effects dampened when requirements risk is high. These findings caution against continuous analysis in outsourced projects while they support joint decision making and continuous integration. The findings also empirically substantiate the largely untested assertion that agile practices help cope with changing requirements.

## Introduction

Information systems (IS) projects have a notorious reputation for running over time and budget while not fully satisfying user needs [1]. Many organizations are therefore turning to agile methods, hoping to increase software quality, reduce costs, shorten time-to-market, and better handle changing priorities by using agile methods [2]. Teams using agile methods typically tailor their use of agile methods [2], i.e., they select the practices to be used in a particular project from the practices advocated in methods such as Scrum [3] and XP [4]. A key question for these teams is which practices are most likely to lead to a successful project given the characteristics of the project at hand.

Over the past two decades, empirical research on agile software development has accumulated knowledge that provides valuable guidance to these teams [5, 6]. Some research has found positive associations between the use of agile methods in general (rather than of particular practices) and project success [7, 8], indicating that the general use of agile methods can enhance project success. Other studies have examined the effects of particular agile practices on project success and found positive effects of pair programming [9, 10] and continuous integration [11] and negative effects of daily stand-ups [10].

Notwithstanding these insights, evidence of the links between agile practices and project success remains limited in three major ways. First, in contrast to practices that involve engineers only (e.g. pair programming, continuous integration) [12], less is known about the impact of *practices that involve engineers and business people*, such as joint decision making and continuous analysis. While qualitative research has explored such practices, quantitative evidence of their effect on project success is scarce [13]. Such evidence could help practitioners navigate the tradeoff between the benefits (e.g. enhanced feedback and communication) and drawbacks (high search costs, opportunistic threats) associated with these practices. Second, few studies have examined the effects of particular agile practices in the context of *outsourced IS projects*, i.e., in settings where client organizations delegate development work to external vendors. This is problematic because not all agile practices may work equally well across firm boundaries [14, 15]. For instance, frequent requirements revision and reprioritization can entail high contract adaption costs and opportunistic behavior in outsourced projects. Third, there is surprisingly little evidence of the context factors under which particular agile practices are effective. In particular, we lack evidence of the potential moderating role of *requirements risk* (i.e., the degree to which requirements are uncertain and frequently changing) [1] in the relationship between agile practices and project success despite the frequent claim that agile methods help cope with changing requirements.

This paper theorizes and empirically examines how three agile practices affect the success of outsourced software projects and how these associations are contingent on requirements risk. The three agile practices in the focus of the paper are continuous integration (compiling, building, deploying, and testing code several times a day), continuous analysis (continuously triggering and incorporating new information about requirements), and joint decision making (client and vendor making important decisions jointly). Drawing on a perspective of software development as knowledge integration, it is argued that practices for knowledge integration within the vendor (continuous integration) come primarily with benefits whereas practices for client-vendor knowledge integration (continuous analysis, joint decision making) come with both benefits and costs. As such the overall effects of client-vendor practices will depend on the need for knowledge integration, which is primarily rooted in difficulties to articulate requirements up-front (i.e., requirement risk). Hypotheses derived from these ideas are tested using data collected through a matched survey of 60 client sponsors and 60 vendor engineers. The results emphasize the benefits from within-vendor practices, draw a more differentiated picture of the effects of client-vendor practices, and largely support the often asserted but rarely tested moderating role of requirements risk.

## Theory Background

### Software Development as Knowledge Integration

This paper explains the impact of agile practices on the success of outsourced projects by drawing on the perspective of software development as knowledge integration. A knowledge integration perspective holds that project team members possess and acquire heterogeneous knowledge and that a key challenge lies in fusing this heterogeneous knowledge into usable software [16, 17]. Software development teams typically comprise business people possessing business knowledge (e.g. ideas about requirements for the software) and engineers possessing technical knowledge (e.g. programming languages, design patterns) and knowledge about existing software systems [17–19].

From a knowledge integration perspective, a key challenge lies in the *interdependencies between these knowledge areas*. For instance, business people often realize their requirements (business knowledge) only after they have seen a first version of the software [16, 20]. But engineers can build a first version (i.e., apply their technical knowledge) only after business people articulate a first version of requirements (business knowledge). These interdependencies challenge the assumption of independence of requirements (business knowledge) from design options (technical knowledge) and from the functionality of existing software systems (software knowledge), an assumption inherent to plan-based software development [16, 17]. Interdependencies exist not only between the business and technical spheres but also within spheres. For instances, interdependencies within the technical sphere manifest because engineers need to know about design decisions and code changes made by other engineers to ensure alignment with own code contributions. The strength of these interdependencies is, to large extent, driven by *requirements risk* (i.e., the degree to which requirements are uncertain and frequently changing) [1]. The fuzzier the ideas about requirements are, the more efforts will be needed to arrive at a shared understanding of how a useful software for the given purpose should look like and the more efforts will be needed to coordinate development actions in the face of changing requirements.

Although knowledge integration issues and requirements risk arise in a variety of projects, *outsourced projects* face the peculiar challenge of integrating the client’s business knowledge and the vendor’s technical knowledge *across firm boundaries* [17, 18]. The rich literatures on IS outsourcing and on theories of the firm point to two key challenges that arise from this boundary [21–23]. First, individuals from different organizations often lack shared knowledge and shared assumptions about effective problem-solving processes, which makes coordination more difficult [21, 22]. Second, different organizations may work towards different goals. In particular, fuzzy requirements may invite opportunistic behavior by the vendor because it is difficult to legally enforce contracts when requirements are unclear at the outset [22, 23].

### Agile Practices for Within-Vendor and Client-Vendor Knowledge Integration

Agile methods help address knowledge-related interdependencies by establishing feedback processes and team-based organizing structures [20]. Three agile practices may be particularly suitable to this end: continuous integration, continuous analysis, and joint decision making. While continuous integration addresses within-vendor knowledge integration, continuous analysis and joint decision making address knowledge integration between client and vendor.

#### Within-Vendor Knowledge Integration.

A key agile practice for within-vendor knowledge integration is *continuous integration*, i.e., the practice of engineers compiling, building, and testing code many times a day, typically by relying on tools for automating build and deployment processes [11, 24]. Continuous integration promotes knowledge integration within the technical sphere (i.e., between engineers) because it provides engineers with immediate feedback about how their code contributions work together with other engineers’ contributions [20]. An attractive feature of continuous integration in outsourcing is that it enables rapid feedback without involving the client, allowing the use of the practice even in projects in which clients do not fully embrace agile methods. Although there is evidence linking continuous integration to higher quality and productivity in open-source development [11], there is little research examining whether these benefits also hold in outsourcing.

#### Client-Vendor Knowledge Integration.

A key agile practice for client-vendor knowledge integration is *continuous analysis*, i.e., continuously triggering and incorporating new information about requirements. The notion of triggering information about requirements alludes to the fact that business people often lack clarity about their requirements at the outset of projects and trigger this information through activities such as testing early versions of the software. They incorporate this information by revising or reprioritizing requirements for the next iteration. In Scrum, continuous analysis manifests through the revision and reprioritization of the product backlog during sprint planning and through the test of the software in the sprint review [3]. Continuous analysis is in line with the agile manifesto principles to “[w]elcome changing requirements, even late in development” and to “[d]eliver working software frequently” [25]. It encompasses concepts such as iterative requirements engineering [26], dynamic prioritization [27], agile requirements prioritization [13, 15], and iterative requirements [13]. Continuous analysis enables client-vendor knowledge integration because it establishes a feedback loop between requirements that result from the business knowledge primarily held by the client and the working version of the software that results from the technical knowledge primarily held by the vendor [17]. Although this feedback loop may be particularly valuable for addressing the lack of shared understanding in outsourcing, continuous analysis may also entail contract adaption costs and opportunistic threats in outsourcing, as I will argue later. Given this trade-off, an important unresolved challenge for practitioners is to decide on the amount of analysis that is made up-front versus continuously throughout a project [28].

A second key practice for client-vendor knowledge integration is *joint decision making* [29], defined as the extent to which important decisions are jointly made by client and vendor. In Scrum, joint decision making manifests in decision making by a team that comprises not only engineers but also the product owner as a business representative [3]. Joint decision making is related to the agile manifesto principle that “[b]usiness people and developers must work together daily throughout the project” [25]. Although daily interaction with business people may not always be possible in outsourced software projects, it is possible to frequently interact in order to make important decisions jointly, which is akin to a team-based organizing mode [14]. Joint decision making promotes knowledge integration between client and vendor because it urges each party to communicate and to incorporate the other party’s perspective when making important decisions. While joint decision making may thus help address the frequent lack of shared knowledge in outsourcing, it may also entail lower benefits of specialization and opportunistic threats, as I will argue later. An important unresolved issue is thus under which circumstances projects should leverage joint decision making versus an approach where clients make business decisions and vendors technical decisions [28].

## Hypotheses

Drawing on the knowledge integration perspective outlined above, this section develops hypotheses about how the three practices and their interaction with requirements risk affects project success. In line with prior studies on IS project success [30, 31], the focus lies on two dimensions of success: effectiveness and efficiency. Effectiveness (also termed product performance) refers to the degree to which the developed software meets the client’s requirements whereas efficiency (also termed process performance) refers to the extent to which a project is completed within time and budget [31].

### Continuous Integration

Continuous integration is likely to enhance success by enabling rapid feedback within the vendor’s development team and efficiency gains due to automation. By frequently compiling, deploying, and testing software, vendor engineers receive rapid feedback on their code contributions, allowing the early discovery of integration problems (within-vendor knowledge integration). Identifying defects early has positive impact on the quality of the delivered software (i.e., effectiveness) because it will make it easier for developers to fix defects before go-live. Continuous integration will also have positive impact on efficiency because problems are identified more easily when only small code contributions are added at a time and because the automation infrastructure behind continuous integration shortens work and wait times [32]. In outsourcing continuous integration appears particularly suitable because it allows leveraging feedback processes irrespective of the degree to which clients are willing to adopt agile practices.

While continuous integration is likely to have a positive main effect on project success, this effect will be more pronounced under high requirements risk. When requirements are uncertain and frequently changing, this has downstream effects by making the engineers’ work more uncertain, increasing thus the need for knowledge integration. Continuous integration will help address this increased need by providing engineers with rapid feedback on their code contributions. These arguments suggest:

- *H1a: Higher amounts of continuous integration are positively associated with success (i.e., effectiveness and efficiency).*
- *H1b: The association between continuous integration and success depends on requirements risk such that the association is stronger when requirements risk is high.*

### Continuous Analysis

Unlike continuous integration, continuous analysis presents projects with a trade-off between the benefits and the costs that arise from the practice. Continuous analysis enables client-vendor knowledge integration by allowing clients to learn about requirements and their relative importance when looking at new versions of the software and discussing requirements with the vendor [13, 26]. In line with these ideas, a case study reported increased client satisfaction due to continuous analysis [33].

Notwithstanding these benefits, continuous analysis practices are also associated with two caveats. First, continuous analysis may involve a long and costly search process where business people realize their true requirements only after developers have spent high efforts on developing functionality that ends up discarded. In outsourced projects, these search processes can also entail high efforts for adapting contracts [14]. Second, frequently revising requirements introduces opportunistic threats in outsourced projects [23]. Vendors may opportunistically leverage the fuzziness of initial specifications to ask for generous compensation of work that was not originally anticipated.

Given these benefits and drawbacks, it is difficult to predict the net effect. However, it is likely that the benefits and drawbacks are salient to a different degree depending on requirements risk. Under high requirements risk, it may not be feasible to accurately identify requirements during a detailed up-front analysis [16]. Continuous analysis will then often be the only feasible alternative. Conversely, when requirements risk is low, articulating requirements up-front is feasible and disciplined up-front analysis may be more efficient that continuous analysis. This leads to the following hypothesis:

- *H2: The association between continuous analysis and success depends on requirements risk such that the association is more positive when requirements risk is high than when requirements risk is low.*

### Joint Decision Making

Like continuous analysis, joint decision making presents projects with a trade-off between benefits and drawbacks of the practice. If client and vendor make key decisions jointly, this entails high amounts of communication, which allows the different stakeholders to integrate their knowledge and may lead to higher project success [34, 35]. Indeed, studies of agile software development point to the importance of close customer collaboration [36] and of reconciling the perspectives of all participants [37].

Notwithstanding these benefits, joint decision making comes at the costs of sacrificing economies of specialization and of opportunistic threats. Economies of specialization may be sacrificed because, as indicated by the knowledge integration literature, it can be difficult and effortful to transfer knowledge from one domain to another [17, 38]. From this perspective, joint decision making can involve high communication efforts and the risk that the voice of the person most knowledgeable in a domain is overruled by others. A potentially more efficient alternative can be to leave business decisions to the client and technical decisions to the vendor. Joint decision making may also entail opportunistic threats because vendors may, for example, falsely attribute a problem in the software to a joint client-vendor decision rather than to their own omissions.

Like in the case of continuous analysis, the net effect of these benefits and drawbacks is unclear. It is likely, though, that the relative size of these benefits and drawbacks depends on requirements risk. When requirements risk is low, the need for knowledge integration is low. It is then feasible for the client to make business decisions and for the vendor to make design decisions based on the client’s business decisions [17]. With each party making decisions in the area in which the party is most knowledgeable, this approach will ensure efficiency and accountability [14, 17]. Conversely, when requirements risk is high, this separation of decisions rights may not be feasible because clients will make poor decisions about requirements at the outset, and design decisions based on poor requirements are unlikely to yield a satisfactory software. I therefore anticipate:

- *H3: The association between joint decision making and success depends on requirements risk such that the association is more positive when requirements risk is high than when requirements risk is low.*

## Methods

### Data Collection

I tested the hypotheses through a matched survey involving client sponsors reporting on project success and vendor engineers reporting on agile practices and further variables. A matched survey addresses concerns of common-method bias [39] and allows gathering data from the informants most knowledgeable about each construct (i.e., sponsors reporting about success, engineers reporting about agile practices). The sampling frame were outsourced IS projects that were completed within the last 12 months. Students and I contacted client organizations from Switzerland and Denmark. Once they agreed to participate, they identified a list of suitable projects along with contact information of the sponsor, the project manager (not used for this study), and a developer from the vendor. We then invited sponsor, project manager, and developer to respond to an online questionnaire that was specifically designed for their role (sponsor, project manager, developer). We obtained responses from 100 sponsors and 92 engineers. Responses matched for 65 projects. From these 65 projects, I removed 5 due to missing data or due to unengaged responses, yielding a final sample size of 60. Table 1 shows sample characteristics. 49 responses (82%) were from the public sector. The sample did not include offshore projects. All projects except for 2 were single-sourcing.

**Table 1.** Sample characteristics

Project size	# Projects	Country	#	Sector	#	Type	#
$0–$100 K	12	Switzerland	40	Public	49	Development	38
$100 K–$1 M	33	Denmark	20	Private	11	Enhancement	22
$ > 1 M	15						

### Instrument Development, Validation, and Estimation

Table 2 shows the final instrument. I relied on existing scales with the exception of the continuous analysis construct for which I developed new items. In line with the definition of continuous analysis as the continuous triggering and incorporating of information about requirements, the items asked about triggering (CA4-5) and incorporating (CA1-3) information, measuring the frequency of these activities in order to capture to what extent they were performed continuously. Following established positivist survey design procedures [40], we performed a pretest with 6 practitioners using an item rating task and a pilot test comprising 43 responses. I used SmartPLS (v3.2.8) to assess the validity of the final instrument. To establish convergent validity, I verified that average variance extracted (AVE) was greater than .50 for all latent constructs (lowest AVE value: .56) [41]. Moreover, all factor loadings were at least .6, with their average exceeding .7 for all constructs [42]. To establish discriminant validity, I verified that construct correlations were below AVE square roots [41]. Discriminant validity was also supported by the HTMT Ratio Test [43]. Reliability was supported by Cronbach alpha values above .7 (see Table 2).

I used OLS regression to estimate the models. OLS regression has higher power for detecting interaction effects than alternative strategies such as PLS or AMOS [44]. The regression models included several control variables. *Task interdependence* reflects the degree to which development team members affect each other in their work [45]. *Knowledge specificity*, a construct from outsourcing research, reflects the degree to which engineers need knowledge specific to client in order to perform their work [46]. Both high task independence and high knowledge specificity might invite the use of agile methods and may correlate negatively with success. It is therefore important to control for these variables. I also controlled for project size, country (Switzerland vs. Denmark), and sector (public vs. private), for similar reasons. As established in social science research, I relied on hierarchical regression, where I first estimated a model with main effects and then added interaction effects. Given the relatively small sample size of 60 (which is largely due to the matched survey design), I considered significant effects at the p < .1 level in the analysis. I verified that the assumptions behind OLS regression were met. Variance inflation factors were below 10 (highest value: 2.67), indicating no concern with multicollinearity. Residual plots were in line with the pattern of a normal distribution. Scatter plots showed no departure from linear effects.

## Results

Table 3 shows the regression results. High R^2^ values ranging from .37 to .50 support the explanatory power of the models. H1a predicted a positive relationship between continuous integration and project success. As the results show, continuous integration had no significant association with effectiveness (β = .18, p > .1, Model 1a) but a significant positive association with efficiency (β=.23, p<.1, Model 1b). H1a is thus supported for efficiency but not for effectiveness. H1b predicted a positive interaction of this relationship with requirements risk. I found a significant positive interaction effect for effectiveness (β = .20, p < .1, Model 2a) and an insignificant interaction effect for efficiency (β = .02, p > .1). H1b is thus supported for effectiveness but not for efficiency.

Even though no main effects of continuous analysis were hypothesized, there was a significant negative effect of continuous analysis (β = −.31, p < .05) on effectiveness. H2 predicted a positive interactive effect of continuous analysis and requirements risk on success. The results show such a positive interaction effect for effectiveness (β = .31, p > .05) but not for efficiency. H2 is thus supported for effectiveness.

**Table 2.** Survey items

Construct	Items	Source
Effectiveness (α = .89)	The software … [Effect1] … meets the functional requirements^a,^ [Effect2] … meets end user requirements^a^ [Effect3] … fulfils technical requirements^a^ [Effect4] … is reliable^a^ [Effect5] … meets expectations with respect to ease of use^a^	[47, 48]
Efficiency (α = .90)	[Effic1] All services were provided on time^a^ [Effic2] The services in this project were provided exceptionally quickly^a^ [Effic3] We ([client]) incurred large unplanned efforts for coordinating and monitoring [vendor] (reverse-coded)^a^ [Effic4] We ([client]) incurred large unplanned efforts for guiding [vendor] (rev.)^a^	[46, 47, 49]
Continuous integration (α = .82)	[CI1] Members of the development team integrate code changes several times a day^a^ [CI2] The development team has a process that generates a build of the software several times a day^a^ [CI3] The developer team is automatically notified of any issues related to the automated compiling, deployment or testing of code^a^ [CI4] In this project, we create the build (i.e., an executable version of the software such as by including configuration files and an installer) in a fully automated way (e.g. by using a script or code)^a^ [CI5] How often does the development team deploy code during development phases to environments to which [client] has no access?^b^	[2]
Continuous analysis (α = .83)	How often do you perform the following actions: [CA1] … Adjust requirements^b^ [CA2] … Evaluate the priorities of requirements^b^ [CA3] … Set the delivery scope for a particular period^b^ [CA4] … Have software tested by employees of [client]^b^ [CA5] How often does the development team deploy code during development phases to environments to which [client] has access?^b^	Newly developed
Joint decision making (α = .76)	In this project, [client] and [vendor] … [JDM1] … set goals together^a^ [JDM2] … developed task strategies together^a^ [JDM3] … diagnosed problems together^a^ [JDM4] … evaluated deliverables together^a^	[29]
Requirements risk (α = .81)	This project was characterized by … [RR1] … continually changing scope and system requirements [RR2] … unclear requirements [RR3] … conflicting requirements [RR4] … requirements not adequately identified	[1]

^a^ 5 point Likert scale (completely disagree, rather disagree, neutral, rather agree, fully agree)

^b^ 7 point scale (less than once a month, once a month, several times a month but not every week, about once a week, several times a week but not every day, about once a day, more often than once a day)

**Table 3.** Regression results

Predictor	Model 1a: *Effectiveness,* main effects	Model 2a: *Effectiveness,* main and interaction effects	Model 1b: *Efficiency,* main effects	Model 2b: *Efficiency,* main and interaction effects
Intercept	−.06 (.28)	.02 (.26)	−.13 (.27)	−.10 (.26)
Task interdependence	−.15 (.13)	−.18 (.12)	−.11 (.13)	−.11 (.12)
Knowledge specificity	**−.24**^**†**^ **(.14)**	−.20 (.13)	−.29* (.13)	−.24 (.13)
Project size	.25 (.20)	.03 (.20)	.27 (.20)	.07 (.20)
Public sector	−.28 (.40)	−.29 (.37)	−.42 (.39)	−.45 (.37)
Switzerland	.43 (.38)	.44 (.35)	**.72**^**†**^ **(.37)**	.83* (.36)
Requirements risk	−.02 (.13)	.00 (.13)	.04 (.13)	.01 (.13)
Continuous integration	.18 (.13)	.18 (.12)	**.23**^**†**^ **(.13)**	**.23**^**†**^ **(.12)**
Continuous analysis	−.31* (.12)	−.31** (.11)	−.17 (.12)	−.17 (.12)
Joint decision making	.17 (.13)	.13 (.12)	**.24**^**†**^ **(.13)**	.20 (.12)
Continuous integration × requirements risk	–	**.20**^**†**^ **(.11)**	–	.02 (.11)
Continuous analysis × requirements risk	–	.31* (.13)	–	.19 (.13)
Joint decision making × requirements risk	–	.15 (.12)	–	.27* (.12)
R^2^	.37	.50	.41	.50
Adj. R^2^	.26	.38	.30	.37
F	3.26** (9, 50)	3.97*** (12, 47)	3.83** (9, 50)	3.84 (12, 47)***

(^†^ p < .1, * p < .05, ** p < .01, *** p < .001, n = 60, standard errors in parentheses, significant numbers in bold, all variables standardized except for binary variables)

Although not hypothesized, joint decisions had a significant main effect on efficiency (β = .24, p < .1). H3 predicted a positive interactive effect of joint decision making and requirements risk on success. Such a significant positive effect was found for efficiency (β = .24, p < .1) but not for effectiveness (β = .17, p > .1).

## Discussion

**Fig. 1.**
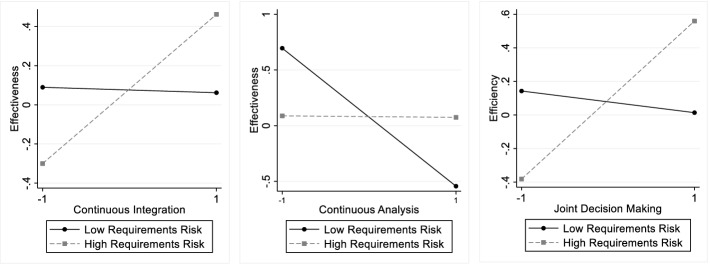
Interaction plots

This research was motivated by a lack of studies that examined how particular agile practices affect the success of outsourced projects and how these effects depend on requirements risk. I found a positive main effect of *continuous integration* on efficiency and a positive interactive effect of continuous integration and requirements risk on effectiveness. The left-hand side of Fig. 1 illustrates this interaction effect. As the plot shows, continuous integration contributes strongly to effectiveness when requirements risk is high (i.e., one standard deviation above the sample mean, see the steep slope of the dashed line) while continuous integration hardly contributes to effectiveness when requirements risk is low (i.e., one standard deviation below the sample mean, see the relatively flat solid line). By and large, these findings echo the expectation that teams hardly face a trade-off when deciding for or against continuous integration practices. It appears that the rapid feedback and automation efficiencies associated with continuous integration make it easier for teams to deliver software on time and in budget. Moreover, when requirements are uncertain and frequently changing, continuous integration helps teams to develop software of high quality despite a volatile environment, as indicated by the positive interaction effect on effectiveness. These findings echo Vasilescu and colleagues’ [11] observation that continuous integration led to higher quality and productivity in open-source software development while the findings extend the boundary conditions of this effect to outsourced projects.

I found no significant effect of *continuous analysis* on efficiency and a significant negative effect on effectiveness, which is dampened under high requirements risk. The interaction plot in the center of Fig. 1 illustrates this interaction. As the plot shows, continuous analysis has a strong negative relationship with effectiveness under low requirements risk (see the negative slope of the solid line) and hardly any effect on effectiveness under high requirements risk. These results indicate that, in outsourced projects, the drawbacks from continuous analysis dominate over its benefits, in particular in projects with low or moderate requirements risk. It seems that continuously revising and reprioritizing requirements based on the insights gained from testing the software is jeopardizing the quality of the software, unless in settings where requirements are highly uncertain. Possibly, continuous analysis results in search processes where engineers spend high efforts addressing requirements that turn out not to be needed, giving thus engineers too little time to develop the features that are needed. It might also be that vendors opportunistically shirk efforts when continuous analysis has eroded the accountability required for contractual governance. While these findings resonate with the classic finding that scope creep jeopardizes project success [31], they suggest that the positive effects of incorporating ongoing customer learning found in other settings [13, 20, 33] need not necessarily transfer to outsourced projects, where the interface between engineers and business people is complicated by firm boundaries. It may also be that the negative results on continuous analysis reflect the fact that 82% of the projects were from the public sector, an environment where public tendering procedures can make it difficult to deviate from initial specifications [50].

I found no significant effect of *joint decision making* on effectiveness but a significant positive effect on efficiency, which is even stronger when requirements risk is high. The interaction plot on the right-hand side of Fig. 1 illustrates this interaction. While joint decision making hardly has an effect on efficiency when requirements risk is low, it has a strong positive effect when requirements risk is high. This suggests that clients and vendors should make important decisions jointly in those projects where at least moderate amounts of uncertainty is surrounding software requirements. Under these circumstances, joint decision making may help ensure that both economic and technical concerns are taken into account when problems or modified requirements call for new decisions to be made. These findings are in line with the benefits from tight customer collaboration and frequent communication found elsewhere [35–37] although my findings also show that the benefits from joint decision making fade to the extent that requirements become more certain. Indeed, when requirements are well known, a more classic division of decision making where the client makes business decisions and the vendor technical decisions can be slightly more efficient according to the results.

Importantly, although both continuous analysis and joint decision making are complicated by firm boundaries in outsourcing, the results indicate that joint decision making is beneficial while continuous analysis is not. Possibly joint decision making can better address the opportunistic threats inherent to outsourcing than continuous analysis because it allows clients and vendors to blend their knowledge while also helping to develop cooperative norms and giving clients control over development work without sacrificing the accountability enabled by clear up-front requirements.

### Contributions

This study makes three key contributions. First, it contributes to the discourse on agile practices in *outsourced projects*. While existing work on outsourcing has provided case study evidence [15] and developed arguments centered on geographic dispersion [14], this paper extends existing work by providing quantitative evidence of the effects of practices on success and by incorporating arguments of the theory-of-the-firm literature, which focuses on opportunistic threats and knowledge barriers due to firm boundaries. The findings reported here echo Batra’s [14] expectation that continuous integration (or delivering working software frequently) is effective in outsourcing while continuous analysis (or welcoming changing requirements) can be problematic. These findings are also consistent with our expectation that continuous feedback processes within the vendor team (i.e., continuous integration) are less problematic than continuous feedback processes that involve client and vendor (i.e., continuous analysis). Extending Batra’s expectation that joint decision making (or business people and developers working together daily) is difficult to enact, the results show that joint decision making can contribute to project efficiency, making this a prime strategy for client-vendor knowledge integration under the opportunistic threats associated with outsourcing. Taken together, a key practical recommendation for outsourced projects is to engage in a detailed up-front analysis akin to plan-based software development (i.e., low use of the continuous analysis practice) involving both client and vendor, while leveraging continuous feedback during development through continuous integration practices.

Second, the paper contributes to the discourse on agile practices that involve engineers and business people. While research on continuous requirements engineering has produced important insights into *how* teams can best enact continuous analysis and joint decision making [13, 15, 26], the study at hand contributes evidence of the effects of these practices on project success, and thus implications for *whether* teams should rely on these practices in a given project. Indeed, important unresolved challenges for practitioners are to decide on the amount of analysis that is made up-front versus continuously throughout a project and on the extent parties from all business and technical domains should be involved in decision making [28]. These questions are gaining importance as agile practices are increasingly used in enterprise-level projects where organizations blend agile and plan-based practices to balance the needs for control and flexibility [28, 51]. Although the findings obtained here on outsourced projects need not generalize to other settings, they point to the potential caveats of business-facing practices, in particular continuous analysis. Moreover, this paper shows a research design that allows empirically evaluating business-facing agile practices in other settings.

Third, this study provides some empirical justification for the largely untested assertion that agile methods help cope with changing requirements. The results demonstrate that all three agile practices have more positive effects (either on effectiveness or on efficiency) when requirements risk was high. This is important evidence for teams wishing to select the practices most likely to increase the success of a project at hand.

### Strengths and Limitations

The study presented here has strengths and limitations. A strength is the matched survey design to avoid common-method bias, which is otherwise often difficult to rule out in survey research. A drawback of this approach was the low sample size, which implied relatively low power. Another strength of the paper is the relatively high variance explained (R^2^ values) due to the use of control variables (e.g. knowledge specificity) that have high explanatory power and that have rarely been used IS project research. Yet, despite the use of powerful control variables, the correlational research design does not allow ruling out endogeneity due to self-selection of agile methods. Future research could rely on econometric techniques to allow stronger causal inference. Another limitation is the sample, which is characterized by a high percentage of projects from the public sector. Future research could examine whether the findings hold in sample with more projects from the private sector. Finally, this study examined the moderating role of requirements risk but not of other potentially relevant factors such as geographic distance, project size and type, the client’s agile culture, and the sourcing design (e.g. multi-sourcing [52], plural sourcing). This remains future research.
